# Exome functional risk score and brain connectivity can predict social adaptability outcome of children with autism spectrum disorder in 4 years’ follow up

**DOI:** 10.3389/fpsyt.2024.1384134

**Published:** 2024-05-16

**Authors:** Tingting Luo, Manxue Zhang, Sixun Li, Mingjing Situ, Pei Liu, Meiwen Wang, Yujie Tao, Shengnan Zhao, Zhuo Wang, Yanping Yang, Yi Huang

**Affiliations:** Mental Health Center, West China Hospital of Sichuan University, Chengdu, China

**Keywords:** autism spectrum disorder (ASD), exome functional risk score (EFRS), brain connectivity, functional connectivity (FC), individual differential structural covariance network (IDSCN), outcome, social adaptability, prediction

## Abstract

**Introduction:**

Autism Spectrum Disorder (ASD) is a common neurodevelopmental disorder emerging in early childhood, with heterogeneous clinical outcomes across individuals. This study aims to recognize neuroimaging genetic factors associated with outcomes of ASD after a 4-year follow-up.

**Methods:**

A total of 104 ASD children were included in this study; they underwent clinical assessments, MRI data acquisition, and the whole exome sequencing (WES). Exome functional risk score (EFRS) was calculated based on WES; and two modalities of brain connectivity were constructed based on MRI data, that is functional connectivity (FC) for functional MRI (fMRI), and individual differential structural covariance network (IDSCN) for structural MRI (sMRI), to explore the neuroimaging genetic biomarker of outcomes of ASD children.

**Results:**

Regression analysis found EFRS predicts social adaptability at the 4-year follow-up (Y = -0.013X + 9.29, *p* = 0.003). We identified 19 pairs of FC associated with autism symptoms severity at follow-up, 10 pairs of FC and 4 pairs of IDSCN associated with social adaptability at follow-up, and 10 pairs of FC associated with ASD EFRS by support vector regression (SVR). Related brain regions with prognostic predictive effects are mainly distributed in superior frontal gyrus, occipital cortex, temporal cortex, parietal cortex, paracentral lobule, pallidum, and amygdala for FC, and temporal cortex, thalamus, and hippocampus for IDSCN. Mediation model showed that ASD EFRS affects the social communication of ASD children through the mediation of FC between left middle occipital gyrus and left pallidum (RMSEA=0.126, CMIN=80.66, DF=42, *p*< 0.001, CFI=0.867, AIC=152).

**Discussion:**

Our findings underscore that both EFRS and brain connectivity can predict social adaptability, and that brain connectivity serving as mediator in the relationship of EFRS and behaviors of ASD, suggesting the intervention targets in the future clinical application.

## 1 Introduction

Autism Spectrum Disorder (ASD) is a common neurodevelopmental disorder that has its onset in early childhood, characterized by social communication deficits and restricted, repetitive behavior patterns, which severely affect individual’s daily function and can bring tremendous burden to families and society (1–3). The globally estimated prevalence of ASD is 1%, showing a growing trend by year (4). Recently, the Autism and Developmental Disabilities Monitoring (ADDM) Network reported that 1 in every 36 (2.8%) 8-year-old children in US were found to have ASD (5).

The outcomes of ASD show a broad spectrum of characteristics. Traditionally, ASD has been regarded as a neurodevelopmental disorder whose impact can be profound, severely affecting the quality of life; few of them live alone, have close friends, or permanent employment; the majority require lifelong management and support (6–8), 74% of ASD adults having severe social difficulties (6), 58% having poor outcome (7). However, evidence also suggests that 0–37% of adults or children with ASD have stable sociability, and even no longer meet the diagnostic criteria for ASD (9–13). Predictors of recovery include relatively high intelligence, receptive language, verbal and motor imitation, adaptive skills, and earlier age of diagnosis and treatment (10–12). The heterogeneity of outcomes implies heterogeneity of biological underpinnings of prognosis in ASD, and identifying specific biological markers that affect the outcomes of ASD thus to implement targeted intervention in early days is crucial for ASD.

ASD is a highly heritable disorder, rare variants of large effect size as well as small effect common gene variants all contributing to ASD risk (14–16). Polygenic risk score (PRS) (17) is a statistical tool used in genetic research to estimate an individual’s genetic risk for a particular trait especially for complex disorders such as ASD. It is calculated by summing up the weighted contributions of multiple genetic variants across the genome (18). Its application in psychiatric disorder research has facilitated the identification of individuals at higher genetic risk for developing conditions such as schizophrenia (19, 20), ASD (21–23), attention deficit hyperactivity disorder (24, 25) and major depressive disorder (26). Furthermore, PRS can also provide prediction value on the outcome of ASD, for example, researchers found burden of PRS is significantly high in adult ASD patients with sustained need for specialist care (27). However, few studies have investigated the social adaptation ability of ASD using PRS in the longitudinal study design. In this study, we aimed to employ exome-based functional risk score, calculating a polygenic risk score by using information from exons in the genome, to explore the relationship between gene and social adaptability of children with ASD.

Magnetic resonance imaging (MRI) can facilitate understanding of how the brain structurally and functionally develops differently in people with ASD, although, to date, MRI results in ASD are not conclusive (28). Evidences suggested abnormal growth in the cortical surface between 6 and 12 months of age and greater brain volume between 12 and 24 months of age in children who were later diagnosed with ASD, compared with those not diagnosed with ASD (29). Emerson (30) demonstrated that FCs of 6-moth-old infants with a high familial risk for ASD could predict the diagnosis of ASD at 24 months of age. Moreover, neuroimaging data can also provide prediction value for outcome of ASD, for example, in our previous study (31), we compared the baseline brain white matter differences among ASD with different outcomes in a 4-year followed up design, and found that ASD with optimal outcome exhibited lower fractional anisotropy (FA) in the left superior thalamic radiation (STR.L) than those with negative outcome, indicating that FA value of the STR.L was a significant predictor for outcome of ASD. However, knowledge about the relationship between the brain connectivity in fMRI and sMRI modalities and outcome situations in children with ASD is yet unclear. Collectively, these studies suggest that ASD share disrupted neural pathways which occurred even before the emergence of behavioral symptoms and might provide clues about the outcome of disorder.

However, due to the methodology difficulty in processing massive amounts of data, evidence is still lacking on combined prediction of neuroimaging information with genetic data on the prognosis of ASD. Machine learning (ML) approaches have their unique advantages in dealing with massive amounts of data, especially by integrating neuroimaging data with multiple modalities. As the most widely used ML approach in the detection of ASD, support vector machine (SVM) has presented summary sensitivity and specificity estimates above 76% (32). Support vector regression (SVR), as the extended algorithm of SVM, offers an opportunity to assess the value of brain imaging for predicting ASD behaviors dimensionally. However, study about the outcome prediction of ASD using SVM or SVR is limited at present.

In this 4-year followed up study, we acquired polygenic risk scores, brain connectivity, and outcomes of children with ASD, aiming to: (1) explore the relationship between genetic risk and outcomes of ASD; (2) exam brain regions with predictive effects, identifying reliable indicators for the outcomes of ASD; (3) elucidate the relationships among EFRS, brain and behaviors of ASD.

## 2 Materials and methods

### 2.1 Study design

Participants were recruited, screened, and assessed at West China Hospital of Sichuan University. The research protocol was approved by the Medical Ethical Committee of West China Hospital of Sichuan University, and parents provided written informed consent after receiving a detailed description of the study. Data were used for research purposes only.

182 individuals with ASD were included in this study, all of whom diagnosed by one professional child psychiatrist based on the Diagnostic and Statistical Manual of Mental Disorders, Fifth Edition (DSM-5)(1). Participants were excluded if they met any of the following criteria: (1) neurological disorders, such as epilepsy, encephalitis; (2) intelligence quotient< 70; (3) history of craniocerebral injury; (4) monogenetic diseases, such as fragile X syndrome, tuberous sclerosis, and Rett syndrome; (5) taking psychiatric medications during assessment. The common comorbid disorders such as attention deficit hyperactivity disorder, tic disorders and emotional disorders are not the exclusion criteria. After excluding ineligible subjects, 104 participants remained. Details of participants enrolled in this study and the study process had also been described in our previous published paper (31).

**Figure 1:**
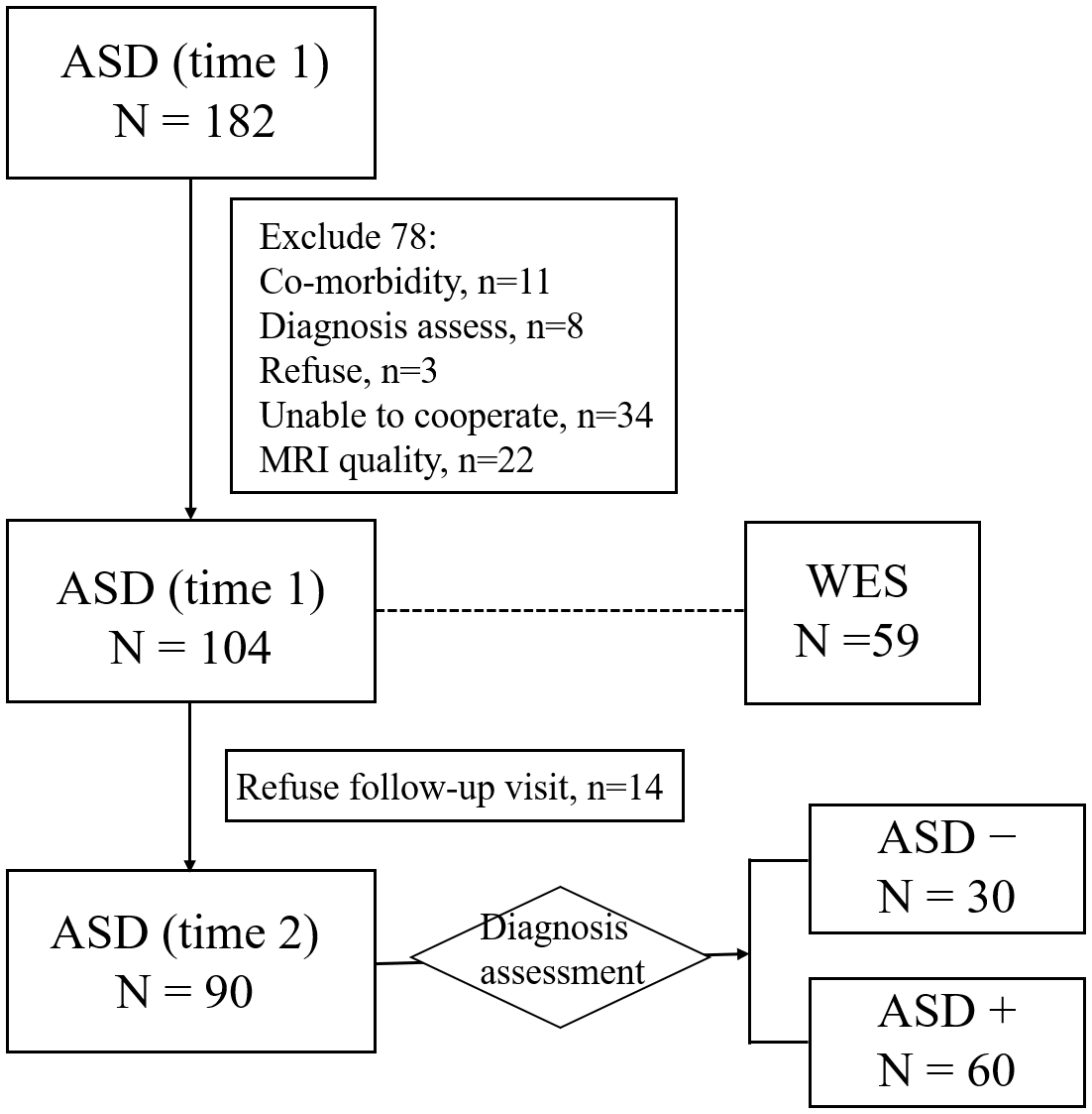
Flowchart for the participant recruitment. ASD−, ASD with optimal outcome; ASD+, ASD with poor outcome; WES, whole exome sequencing.

After recruitment, a parent interview and a child assessment were conducted using the autism diagnostic interview-revised (ADI-R) (33) and the autism diagnostic observation schedule (ADOS) (34) respectively to confirm ASD diagnosis. At baseline (time 1), all participants were required to take an intelligence quotient (IQ) test and have an MRI scan after receiving their diagnoses, if available, collecting blood of participants and their family members. In this study, 59 blood samples of ASD were collected. Around 4 years after enrollment (time 2), participants were requested to take part in a follow-up assessment. In this study, 90 children with ASD completed the follow-up assessment, according to ADOS total score, 30 participants achieving optimal outcomes (ADOS total score< 7) (labeled as ASD-), other 60 participants achieving poor outcomes (ADOS total score ≥ 7) (labeled as ASD+). See **Figure 1** for the flowchart of the participant recruitment.

### 2.2 Instruments

ADI-R, developed and revised by Le Couteur et al. (33), is a standardized, structured interview instrument to evaluate autism symptoms. It consists of four subscales: communication, social interactions, restricted and repetitive behaviors (RRB) and evidence of early developmental abnormalities (35). ADOS, revised by Lord et al. (34), is a standardized structured interactive autism symptom rating tool revised. Individuals were assessed by observing the ASD-related symptoms during the game interaction. It consists of 4 subscales: communication, social interaction, imagination/creativity, and RRB. The total score greater than or equal to 7 is the diagnostic threshold. It is the most used “gold standards” for the diagnosis of ASD in clinical and scientific research, which was also used as a criterion for the severity of symptoms in follow-up assessment of ASD children in this study.

The Chinese-Wechsler Intelligence Scale for Children (C-WISC) (36) was used to assess the intellectual development. The full-scale intelligence scale includes 11 sub-tests. In this study we employed 4-in-1 short version recommended in the appendix of the manual, including knowledge, comprehension, picture completion, and block drawing.

Infants-Junior Middle School Students’ Social-Life Abilities Scale (S-M) (37) and overall social outcome (OSO) ratings (6, 7) were used to evaluate the social adaptability of participants. The S-M scale consists of 132 questions in six domains. After the completion of the questionnaire, a standardized score based on age was obtained. Standard scores ranged from 5 to 13, and the lower the scores, the worse the social adaptability. The OSO scoring system was derived from Rutter’s non-specific scoring criteria for the outcome of psychiatric disorders (38), which introduced operational scoring rules and focused on the domains of independent living, friendship and career (6, 7). Finally, the social adaptability of individuals was graded trichotomy as very good/good, average, and poor/very poor.

### 2.3 Blood collection and the whole exome sequencing

Peripheral blood of 59 ASD subjects and their parents was collected, stored in the refrigerator at -20°C, and regularly sent to the laboratory for DNA extraction. The extracted DNA was stored in a refrigerator at -80°C and sent for the whole exome sequencing (WES).

The main steps of sequencing were as follows: 1) Quality inspection: The quality of DNA samples was tested to detect whether there was obvious DNA degradation and whether there was RNA and protein contamination; 2) Library construction: DNA samples with content above 0.6ug of Agilent SureSelect Human All Exon V6 liquid capture system were effectively enriched to create sequence libraries; 3) Sequencing: high-throughput and deep sequencing was performed on the Illumina HiSeq 4000 platform (Illumina, Inc., San Diego, CA, USA). The preprocessing steps of WES data included extraction, quality control and typing annotation. Details are shown in **Supplementary Materials**. We focused on the polygenic risk of rare mutations, so only mutations with minor allele frequency (MAF)< 0.01, according to the east Asians from the 1000 Genomes Project database (N=504) (39) and the Exome Aggregation Consortium (ExAC) database (N ~= 3000) (40), were retained for subsequent analysis.

### 2.4 Exome functional risk score

Polygenic risk score is a comprehensive assessment of the cumulative effects of multiple variants with weak effects on related diseases to evaluate the genetic risk of developing a disease (41). According to the procedure recommended by PRSice-2, the additive model was used to calculate the exome score (42). We followed the approach developed by Chiara Fabbri (43) to calculate ASD EFRS based on the WES data, and to obtain the load scores of the whole exome rare variants. The EFRS is calculated using the following formula:


$$\sum_{1}^{n}v_{all}*w_{s}*w_{f}$$

where n is the number of genetic variants within the whole exome, v_all_ is the number of alternative alleles, w_s_ is the corresponding functional score of the gene variant, and w_f_ is the frequency weight for that variant. By weighting function and frequency simultaneously, EFRS does not depend on the presence of individual variants which could not be observed in some of the tested samples, and thus keeps the final score stable and reliable (44). Different sources (LRT, Mutation Assessor, Polyphen-2, SIFT and CADD (45–48), see **Supplementary Materials**) were tested in this study, to determine the damaging of mutations for functional scores (w_s_). The frequency weighting (w_f_) was determined based on the mean frequency of east Asian populations in the 1000 Genomes Project (https://www.internationalgenome.org/) and ExAc databases (http://exac.broadinstitute.org). The alternative alleles (v_all_) were determined based on the mutation sites of ASD identified in a WES study of 175 trios published in Nature in 2012 (49).

### 2.5 Image acquisition

All MRI data were collected on a 3T scanner (Philips, Achieva, TX, Best, The Netherlands) at the Tibet Chengban Branch of Sichuan University West China Hospital. Two modalities [structural MRI (sMRI) and resting state fMRI (rs-fMRI)] images were acquired.

sMRI (T1 weighted) images were scanned using a three-dimensional spoiled gradient recalled echoing planar imaging sequence. Detailed scan parameters are described as follows: repetition time, 8.2 msec; echo time, 3.8 msec; flip angle, 7°; slice thickness, 1 mm; field of view, 256 mm × 256 mm; matrix size, 256 × 256; voxel size, 1 × 1 × 1 mm^3^. Bold signals from the rs-fMRI modality were acquired using a single-excitation gradient echo planar imaging (EPI) sequence with scanning parameters described as follows: echo time, 30ms; repeat time, 2000ms; flip Angle, 90°; FOV = 240mm×240mm; slice thickness, 4mm; gap, 0mm. Each time point was scanned continuously, and a total of 240 time points were acquired.

### 2.6 MRI data preprocessing and brain connectivity network construction

Raw data acquired from the MRI scanner (DICOM files) were converted from raw DICOM files into analyzable NIfTI images using dcm2niigui software. Use the SPM software package in MATLAB R2013b platform (https://www.fil.ion.ucl.ac.uk/spm/software/spm12/), FreeSurfer package (https://www.freesurfer.net/) and FSL software (http://surfer.nmr.mgh.harvard.edu/fswiki/Fsl) to analyze imaging data processing.

The whole brain functional connectivity (FC) analysis method based on correlation analysis proposed by Salvador (2005) was used for the construction of FC network (50). Individual differential structural covariance network (IDSCN) was constructed to analyze the common structural changes among the brain regions. Details of data preprocessing and description of the construction of the connectivity networks are provided in the **Supplementary Materials**.

### 2.7 Statistical analysis

Firstly, through simple logistic regression analysis, we examined the relationship between EFRS and outcomes situations (autism symptoms severity and social adaptability). Then, based on the brain connectivity networks, we used ML method SVR to identify the brain regions related to the outcomes of ASD. And then, we explored the relationships among EFRS, brain and outcomes, through multivariate logistic regression analysis with *post hoc* Bonferroni correction, significance level 0.05. Finally, taking the targeted brain regions as a mediator, we constructed a mediation model of “gene-brain-behavioral” to analyze how EFRS affects the behavior of ASD patients through the interaction of brain connectivity. At the same time, imaging genetic biomarkers related to the outcomes of ASD were screened and confirmed.

## 3 Results

### 3.1 Demographic information of participants

After excluding ineligible individuals, there were 104 children with ASD enrolled. The demographic information is shown in **Table 1**. The average age is 8.01(SD = 3.25) years. Four years later after enrollment, 90 individuals were followed up for clinical diagnostic assessment (evaluated by ADOS) and social adaptability (S-M and OSO). Among the 90 individuals, 30 of them (33.33%) showed optimal outcome (labeled as ASD-), whose ADOS total scores less than 7, losing the diagnoses of ASD; while the other 60 subjects’ diagnoses of ASD persistent (labeled as ASD+). Independent sample t test was employed to identify the clinical differences between ASD- group and ASD+ group at baseline (Time 1), and the results showed that there is no difference in age, IQ, gender and ADOS scores (all p > 0.05). While at follow-up (Time 2), significant differences are found in IQ, ADOS, S-M and OSO between ASD- group and ASD+ group (all *p*< 0.001) (see **Table 1**).

**Table 1:** Demographics and clinical characteristics of the participants with different outcomes at baseline and 4 years later.

	ASD	Time 1				Time 2			
	N = 104	ASD- N=30	ASD+ N=60	t/χ^2^	*p* value	ASD- N=30	ASD+ N=60	t/χ2	*p* value
**Age** (years)	7.62 ± 3.60	8.00 ± 4.18	7.46 ± 3.34	0.66	0.511	12.37 ± 4.19	11.48 ± 3.38	1.12	0.103
**IQ**	85.50 ± 20.80	87.93± 20.16	87.34± 19.24	0.14	0.893	99.45 ± 15.72	85.16 ± 17.87	3.72	<0.001***
**Gender**(M/F)	92/12	29/1	52/8	2.22	0.136	29/1	52/8	2.22	0.136
ADI-R									
Communication	13.38 ± 5.04	11.48 ± 3.64	14.14 ± 5.50	-2.14	0.036*	–	–	–	–
Social Interaction	16.91 ± 5.78	14.74 ± 4.30	17.78 ± 6.09	-2.18	0.032*	–	–	–	–
RRB	4.54 ± 2.71	4.00 ± 2.45	4.76 ± 2.78	-1.14	0.258	–	–	–	–
development	2.56 ± 1.67	1.87 ± 1.63	2.83 ± 1.62	-2.39	0.019*	–	–	–	–
total	37.40 ± 12.08	32.09 ± 8.56	39.5 ± 12.67	-2.58	0.012*	–	–	–	–
ADOS									
communication	5.72 ± 2.46	5.60 ± 2.61	5.87 ± 2.15	-0.52	0.607	0.43 ± 0.68	3.97 ± 2.20	-8.57	<0.001***
Social Interaction	9.57 ± 2.66	9.30 ± 3.09	9.82 ± 2.83	-0.79	0.431	0.97 ± 1.35	8.03 ± 3.38	-11.00	<0.001***
Imagine	1.51 ± 1.11	1.33 ± 1.30	1.70 ± 1.20	-1.33	0.186	0.07 ± 0.25	0.83 ± 0.81	-5.07	<0.001***
RRB	1.73 ± 1.38	1.30 ± 1.09	1.80 ± 1.35	-1.76	0.082	0.37 ± 0.72	1.95 ± 1.76	-4.72	<0.001***
Total	18.23 ± 5.31	17.53 ± 6.19	19.18 ± 5.26	-1.32	0.190	1.83 ± 2.07	14.78 ± 6.62	10.44	<0.001***
**S-M**	–	–	–	–	–	9.37 ± 1.27	8.22 ± 1.42	3.75	<0.001***
**OSO**	–	–	–	–	–	1.43 ± 1.14	5.63 ± 1.47	13.71	<0.001***

*p< 0.05, ***p< 0.001. “-” means no available data. ASD, autism spectrum disorder; IQ, intelligence quotient; ADI-R, autism diagnostic interview-revised; ADOS, autism diagnostic observation schedule; RRB, restricted and repetitive behaviors; S-M, infants-junior middle school students’ social-life abilities scale; OSO, overall social outcome ratings; ASD-, ASD with optimal outcome; ASD+, ASD with poor outcome; Time 1, baseline assessment; Time 2, 4-year follow up assessment.

### 3.2 Association between EFRS and the outcomes of ASD children

We calculated the EFRS of the 59 ASD children according to Chiara Fabbri’s method (43), to explore the genetic liability of the outcomes of ASD children. The EFRS values are shown in **Supplementary Table 1**. Among the 59 individuals with WES data, 50 of them achieved follow-up: 15 individuals (30%) had optimal outcome (ASD-), ADOS total score less than 7; the remaining 35 (70%) had ASD diagnoses persistent with poor outcome (ASD+).

**Figure 2:**
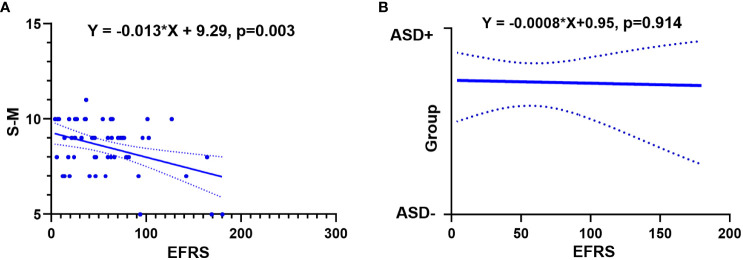
Relationships between EFRS and outcomes of ASD. **(A)** Simple linear regression analysis shows EFRS could predict the social adaptability of ASD children (Y= -0.013*X+ 9.29, p = 0.003); **(B)** Simple logistic regression analysis shows EFRS could not predict the outcome grouping (Y= -0.0008*X+ 0.95, p = 0.914). The solid line shows the distribution trend of the values; The dashed line represents the 95% confidence interval. EFRS, exome functional risk scores; S-M, the infants-junior middle school students’ social-life abilities scale; ASD-, ASD with optimal outcome; ASD+, ASD with poor outcome.

Simple regression analysis showed that EFRS could predict social adaptability of ASD children after 4 years later. When taking social adaptability (evaluated by S-M) as the dependent variable and EFRS as the independent variable, the results demonstrated that EFRS could act as an independent predictor of the social adaptability (Y = -0.013*X + 9.29, *p* = 0.003) (see **Figure 2A**). However, EFRS could not be an independent predictor of the severity of autism symptoms (Y = -0.0008*X + 0.95, *p* = 0.914) (see **Figure 2B**). EFRS was not significantly associated with the total score of ADOS neither at baseline (r = -0.108, p = 0.538) nor at follow-up (r = 0.065, p = 0.653) (see **Supplementary Figure 1**).

### 3.3 Association between brain connectivity and outcomes of ASD children

We built SVR models based on structurally and functionally brain connectivity, IDSCN for structural MRI and FC for functional MRI, to precisely identify the outcome predictors of ASD children. Two candidate indexes were included in the SVR models: 1) symptoms severity: ADOS total score at time 2, and the outcome grouping (ASD+ vs ASD-); 2) social adaptability: S-M standard score and OSO grade at time 2.

**Figure 3:**
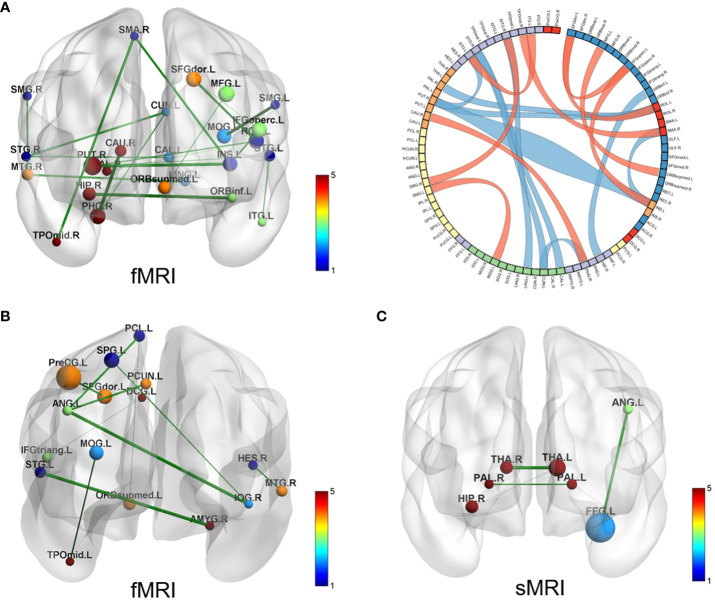
Brain connectivity with predictive effects. **(A)** SVR model for ADOS total scores results based on FC (fMRI); the left displays the 19 pairs of congruent brain regions; the right shows circle diagram of the identified congruent brain regions, red represents positive weight and blue represents negative weight; **(B)** SVR model for social adaptability results based on FC (fMRI), 10 pairs of congruent brain regions displayed; **(C)** SVR model for social adaptability results based on IDSCN (sMRI), 4 pairs of congruent brain regions displayed. SVR, support Vector Regression; ADOS, autism diagnostic observation schedule; FC, functional connectivity; IDSCN, individual differential structural covariance network; fMRI, functional MRI; sMRI, structural MRI.

SVR models for ADOS total scores shown that FC could predict ADOS total scores (the prediction function: Y = 0.30X + 7.67, R^2^ = 0.13, MSE = 79.25, *p* = 0.08) (see **Supplementary Figure 2**). There are totally 19 pairs of FC with predictive effects; 10 pairs of those show negative weight, predicting a decrease in ADOS scores; while the other 9 pairs show positive weight, predicting an increase in the ADOS total scores (see **Figure 3A**). Details of the 19 pairs of FCs are shown in **Table 2**. IDSCN has no predictive effect on ADOS total scores.

**Table 2:** 19 pairs of FC associated with autism symptoms severity.

abbreviation	brain regions	abbreviation	brain regions	weight
ORBinf.L	inferior frontal gyrus, orbital part	HIP.R	right hippocampus	-2.49
INS.L	left insula	PUT.R	right putamen	-2.40
SMA.R	right supplementary motor area	INS.L	left insula	-1.82
LING.L	left lingual gyrus	MTG.R	right middle temporal gyrus	-1.78
CUN.L	left cuneus	STG.R	right superior temporal gyrus	-1.75
CAL.L	left calcarine sulcus	STG.R	right superior temporal gyrus	-1.16
PHG.R	right parahippocampal gyrus	CUN.L	left cuneus	-1.04
ROL.L	left Rolandic operculum	PUT.R	right putamen	-0.97
PUT.R	right putamen	STG.L	left superior temporal gyrus	-0.60
ROL.L	left Rolandic operculum	PAL.R	right pallidum	-0.44
PHG.R	right parahippocampal gyrus	CAU.R	right caudate nucleus	2.65
MOG.L	left middle occipital gyrus	SMG.L	left supramarginal gyrus	2.49
SMA.R	right supramarginal gyrus	TPOmid.R	temporal pole: middle temporal gyrus	2.04
SFGdor.L	left superior frontal gyrus, dorsolateral	ROL.L	left Rolandic operculum	1.88
SMG.R	right supramarginal gyrus	MTG.R	right middle temporal gyrus	1.38
IFGoperc.L	inferior frontal gyrus, opercular part	INS.L	left insula	1.24
STG.L	left superior temporal gyrus	ITG.L	left inferior temporal gyrus	0.91
MFG.L	left middle frontal gyrus	ORBsupmed.L	left superior frontal gyrus, medial orbital	0.77
SFGdor.L	left superior frontal gyrus, dorsolateral	IFGoperc.L	left inferior frontal gyrus, opercular part	0.49

ranking by weight values.

SVR models for social adaptability of ASD children showed that both FC (fMRI) and IDSCN (sMRI) have good predictive effects. The predictive function of FC for the S-M standard score is: Y = 0.42*X + 4.94, R^2^ = 0.13, MSE = 2.33, p = 0.03 (see **Supplementary Figure 2**). A total of 10 pairs of FC were identified to have predictive effects, of which 4 pairs of FCs have positive weight values, indicating that increased FC of these brain regions could predict better social adaptability after 4 years; the other 6 pairs of FC have negative weight values, suggesting that increased FC of these brain regions might predict worse social adaptability after 4 years (see **Figure 3B**, **Table 3**). The predictive function of sMRI for the S-M standard score is: Y = 0.30*X + 5.97, R^2^ = 0.17, MSE = 2.66, *p* = 0.06 (see **Supplementary Figure 2**). A total of 4 pairs of IDSCN were found to have predictive effects with positive weight values (see **Figure 3C**, **Table 3**).

**Table 3:** Brain connectivity associated with social adaptability of ASD children.

abbreviation	brain regions	abbreviation	brain regions	weight
fMRI				
IOG.R	right inferior occipital gyrus	ANG.L	left angular gyrus	1.16
IOG.R	right inferior occipital gyrus	SPG.L	left superior parietal gyrus	0.33
ORBsupmed.L	left superior frontal gyrus, medial orbital	PCL.L	left paracentral lobule	0.10
IFGtriang.L	left inferior frontal gyrus, triangular part	DCG.L	left median cingulate and paracingulate gyri	0.03
AMYG.R	right amygdala	STG.L	left superior temporal gyrus	-1.18
ANG.L	left angular gyrus	PCUN.L	left precuneus	-0.72
PreCG.L	left precental gyrus	SFGdor.L	left superior frontal gyrus, dorsolateral	-0.68
ANG.L	left angular gyrus	PCL.L	left paracentral lobule	-0.67
MOG.L	left middle occipital gyrus	TPOmid.L	left middle temporal gyrus	-0.48
HES.R	right Heschl gyrus	MTG.R	right middle temporal gyrus	-0.45
sMRI				
THA.L	left thalamus	THA.R	right thalamus	0.17
ANG.L	left angular gyrus	FFG.L	left fusiform gyrus	0.15
PAL.R	right pallidum	PAL.L	left pallidum	0.11
THA.L	left thalamus	HIP.R	right hippocampus	0.09

ranking by weight values.

### 3.4 Relationships of EFRS, brain connectivity and outcomes of ASD children

We further tested the extent to which EFRS and brain connectivity identified above analysis could predict outcomes of ASD when taking them together as independent variables. The brain connectivity associated with outcomes found by the SVR models (see **Tables 2**, **3**) and EFRS were included in the multiple regression model to analyze the joint predictive value of the baseline genetic risk and brain imaging characteristics on the outcomes of ASD.

**Figure 4:**
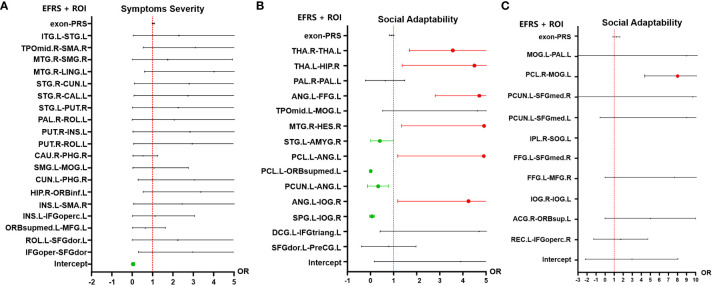
Multiple logistic regression analysis results of outcomes of ASD children. **(A)** Multiple logistic regression results of the symptoms severity (outcome grouping). There is no significant predictive effect of the 19 pairs of FC nor EFRS on symptoms severity of ASD children at 4-year follow-up. **(B)** Multiple logistic regression results of social adaptability (OSO). Risk factors for social adaptability include 3 pairs of IDSCN that is bilateral thalamus, left thalamus (THA.L) to right hippocampus (HIP.R), and left angular gyrus (ANG.L) to left fusiform gyrus (FFG.L), and 3 pairs of FC, that is right Heschl gyrus (HES.R) to right middle temporal gyrus (MTG.R), left angular gyrus (ANG.L) to left paracentral lobule (PCL.L), and left angular gyrus (ANG.L) to right inferior occipital gyrus (IOG.R); protective factors for social adaptability include 4 pairs of FC, that is left superior temporal gyrus (STG.L) to right amygdala (AMYG.R), left paracentral lobule (PCL.L) to medial orbital of left superior frontal gyrus (ORBsupmed.L), left precuneus (PCUN.L) to left angular gyrus (ANG,L), and left superior parietal gyrus (SPG.L) to right inferior occipital gyrus (IOG.R). **(C)** Multiple logistic regression results of social adaptability based on FC associated with EFRS. FC of left middle occipital gyrus (MOG.L) and right paracentral lobule (PCL.R) is a risk factor for outcomes (OR=7.08, 95%CI=5.32 - 12.03). Red represents OR > 1, which is a risk factor for poor outcome; Green represents OR< 1, which is a protective factor for good outcome; Black represents no predictive value; Cut-off values represent OR values much larger than 5. The results are adjusted by *post hoc* Bonferroni correction, and significant p-value is 0.05. IDSCN, individual differential structural covariance network; FC, functional connectivity; EFRS, exome functional risk score; ROI, region of interest.

In the analysis of the autism symptom severity (outcome grouping based on ADOS scores) at follow-up, 19 pairs of FC identified by SVR based on ADOS total score (see **Figure 3A**, **Table 2**) and EFRS were included as predictive variables. The analyses of multiple logistic regression did not identify any brain connectivity that could predict the outcome grouping of ASD children at 4-year follow-up (see **Figure 3A**); In the analysis of social adaptability (OSO grade) at follow-up, the brain connectivity characteristics determined by SVR based on S-M (see **Figure 3B**, **Table 3**) and EFRS were included. The results of the predicting effects of both fMRI (FCs) and sMRI (IDSCN) on social adaptability were verified. As shown in **Figures 4A, B**, 3 of the 4 pairs of IDSCN perform predictive effects, as predictors of poor outcome; 7 of the 10 pairs of FC perform predictive effect, 3 pairs of those as predictors of poor outcome, the other 4 pairs as predictors of good outcome. In conclusion, both FC and IDSCN, the two modalities of brain connectivity could predict the social adaptability of ASD children. The characteristic brain regions mostly located in superior frontal gyrus, occipital cortex, temporal cortex, parietal cortex, paracentral lobule, pallidum, and amygdala for functional brain connectivity, and temporal cortex, thalamus, and hippocampus for structural brain connectivity.

In addition, we employed SVR to identify the characteristic brain regions related to ASD EFRS, to explore the relationship between brain connectivity and EFRS. The SVR models based on FC (fMRI) and IDSCN (sMRI) were constructed taking EFRS as a predictor variable. SVR model construction based on fMRI modality succeeded (regression function: Y = -1.64*X + 154.7, p = 0.05, R = -0.32, MSE = 3168.80), meaning that ASD EFRS has significantly predictive effects on the values of FC; while SVR model based on sMRI failed (regression function: Y = -0.09X + 64.34, *p* = 0.636) (see **Supplementary Figure 3**). There are 10 pairs of FC identified that are associated with EFRS, as shown in **Table 4**.

**Table 4:** ASD EFRS associated with 10 pairs of FC.

abbreviation	brain regions	abbreviation	brain regions
MOG.L	left middle occipital gyrus	PAL.L	left pallidum
MOG.L	left middle occipital gyrus	PCL.R	right paracentral lobule
PCUN.L	left precuneus	SFGmed.L	left superior frontal gyrus, medial
PCUN.L	left precuneus	SFGmed.R	right superior frontal gyrus, medial
IPL.R	right inferior parietal lobule	SOG.L	left superior occipital gyrus
FFG.L	left fusiform gyrus	SFGmed.R	right superior frontal gyrus, medial
FFG.L	left fusiform gyrus	MFG.R	right middle frontal gyrus
IOG.R	right inferior occipital gyrus	IOG.L	left inferior occipital gyrus
ACG.R	right anterior cingulate gyrus	ORBsup.L	left superior frontal gyrus, orbital part
REC.L	left gyrus rectus	IFGoperc.R	right inferior frontal gyrus, opercular part

FC, functional connectivity; EFRS, exome functional risk score.

To further explore the relationship of ASD EFRS and brain regions identified above, and the outcomes of ASD children. We took OSO grade and outcome grouping as dependent variables respectively, and characteristic brain regions related to EFRS (see **Table 4**) as independent variables in the multiple logistic regression analysis, and the results showed that FC of left middle occipital gyrus (MOG.L) and right paracentral lobule (PCL.R) (OR = 7.08, 95%CI = 5.32 - 12.03) is a risk factor for social adaptability of ASD children (see **Figure 4C**). The logistic regression model taking outcome grouping as the dependent variable was not fitted successfully.

### 3.5 Mediation model of EFRS, brain connectivity and behaviors of children with ASD

**Figure 5:**
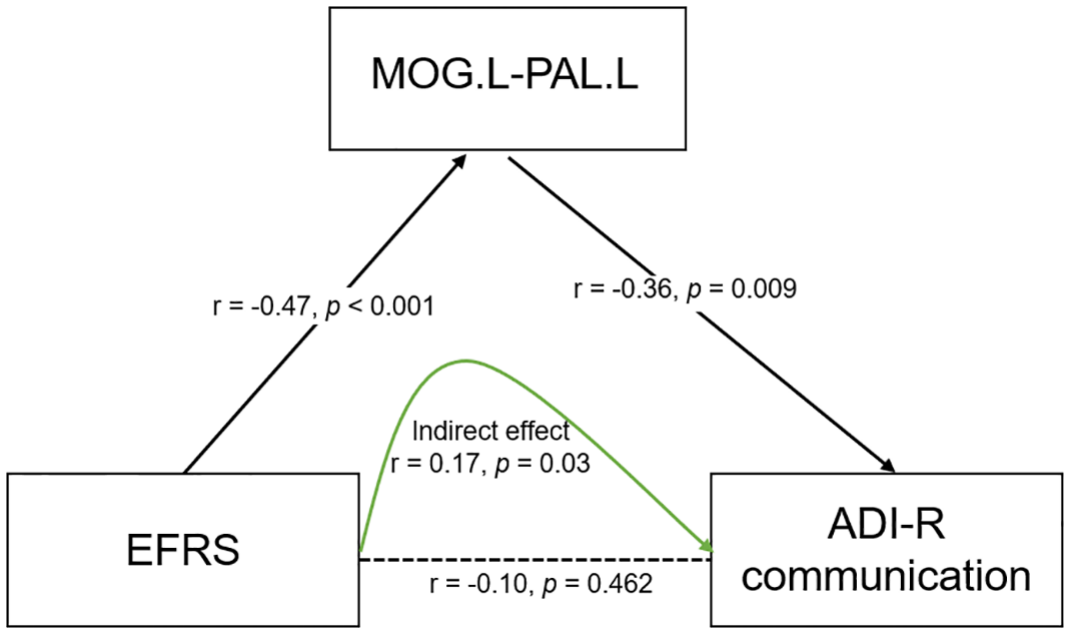
Mediation model of EFRS, brain connectivity and behaviors of children with ASD. EFRS has a direct effect on the FC of MOG.L and PAL.L (r - -0.47, *p*< 0.001); FC of MOG.L and PAL.L has a direct effect on communication symptom (r - -0.36, *p* = 0.009); EFRS has no direct effect on the communication (r = -0.10, *p* = 0.462), but an indirect effect (r = 0.17, p=0.03), through the mediation of FC of MOG.L and PAL.L (CMIN = 80.66, DF = 42, *p*< 0.001, CFI = 0.867, AIC = 152). The black solid line indicates direct effect; the black dashed line indicates the path without significance; the green solid line indicates indirect effect. r is the path weight; EFRS, exome functional risk score; MOG.L- PAL.L, functional connectivity between left middle occipital gyrus and left pallidum; ADI-R communication: communication subscale of the autism diagnostic interview-revised (ADI-R).

To further explore the relationships of EFRS, brain connectivity, and symptoms of ASD children, we constructed a mediation model using structural equation modeling. The results show that EFRS mediates the communication (ADIR subscale) through the FC of left middle occipital gyrus (MOG.L) and left pallidum (PAL.L) (CMIN = 80.66, DF = 42, p< 0.001, CFI = 0.867, AIC = 152), as shown in **Figure 5**. There is no direct effect between EFRS and the communication (r = -0.10, *p* = 0.462), but an indirect effect (r = 0.17, p = 0.03).

## 4 Discussion

This is a 4-year prospective follow-up study of children with ASD, adopted multimodality data like EFRS, brain imaging, and behaviors of baseline and follow up. Firstly, we examined the relationships between EFRS and outcome situations of children with ASD, relationships between brain connectivity and the outcome situations of children with ASD, and relationships between EFRS and brain connectivity, by SVR models, respectively. We built outcome prediction models of ASD children based on EFRS and brain connectivity (FC for fMRI and IDSCN for sMRI), identifying genetic neuroimaging biomarkers of outcomes for ASD children. Finally, we examined the relationships among EFRS, brain connectivity and behaviors of ASD, finding out the way how they interact. In summary, this study found that both EFRS and brain connectivity especially FC show prognostic prediction effects on ASD children, and that EFRS, brain connectivity, and autism symptoms interact, frequently brain connectivity serving as mediator. This study identified candidate brain regions that related to the outcomes of ASD, and found the pathway of which brain mediates the relationship between EFRS and behaviors of ASD children, laying a solid foundation for finding genetic neuroimaging biomarkers for predicting the outcomes of ASD.

Our study has some strengths compared to previous similar studies. First, this is a longitudinal follow-up study, following the outcome situations of children with ASD. Second, we employed EFRS to measure the genetic risk of ASD. Although many ASD-associated *de novo* mutations have been identified through WES, few studies have used exome risk score in ASD. Third, we included two modalities of brain connectivity, IDSCN (sMRI) and FC (fMRI), making the results more credible. In addition, we explored the relationships of polygenic genetic risk, brain imaging, and behaviors of children with ASD, identified the role of brain connectivity; while previous studies mostly examined relationships of two of them. Finally, we adopted SVR algorithm in this study with the consideration that SVR has advantages in dealing with massive amounts of data, thus effectively identifying the genetic imaging factors related to the outcomes of children with ASD.

### 4.1 Outcome situations of children with ASD

In this study, 33.33% of children with ASD achieve optimal outcome, losing the diagnosis for ASD. The rate of recovery is in the range of 0–37% mentioned above (9–13), consistent with the results of previous studies. However, we exclude these ASD children with IQ< 70 and comorbid other neuropsychiatric disorders, meaning that the truly rate of recovery is much lower than 33.33%, thus requiring larger samples to replicate. Children with optimal outcome (ASD- group) also perform better social adaptability than those with poor outcome (ASD+ group), which is consistent with the results of Harstad’ study that 37% of the children who were clinically diagnosed with ASD at 12 to 36 months did not continue to meet diagnostic criteria for ASD at 5 to 7 years of age, and emphasized that the most related factor with the nonpersistent ASD was adaptive skills (12).

We found that ASD EFRS could predict social adaptability of children with ASD 4 years later, suggesting that outcome conditions of ASD children are affected by genetic regulation. A large population-based study from the UK Biobank found that social-isolation polygenic risk score (PRS) predicted friendship at 18 years old, demonstrating that the genetic factors are able to predict related social traits (51), which is agreement with our results. Social adaptability is an important prognostic indicator. A previous study has demonstrated that PRS could predict the severity of ASD (27), while in our study, we failed to draw this conclusion; Our results shown that exome risk score could not predict the severity of autism symptoms, which could be due to the small cohort size of this study.

### 4.2 Relationships between brain connectivity and the outcomes of children with ASD

We identified 19 pairs of FC associated with autism symptoms severity at follow-up (**Table 2**), 10 pairs of FC and 4 pairs of IDSCN associated with social adaptability at follow-up (**Table 3**), and 10 pairs of FC associated with ASD EFRS (**Table 4**) by SVR models. Associated brain regions with prognostic predictive effects are mainly distributed in the extensive cortex regions and pallidum and amygdala of subcortical regions for FC, and the temporal cortex, thalamus, and hippocampus for IDSCN. The big differences between FC and IDSCN may stem from the limited cohort size, while in the other hand, which might reflect the intrinsically heterology in brain connectivity revealed by FC and IDSCN. In our study, FC obtained more significant results than IDSCN, which supports the results of Traut who claimed that functional MRI was more important for prediction than structural MRI (52). One of the reason is that FC reflects the organization and inter-relationship of spatially separated brain regions (53), alteration in brain FC is expected to provide potential biomarkers for classifying or predicting brain disorders. Previously, Guo identified two ASD subtypes based on the inter-individual deviation of FC patterns, which could predict the severity of social communication impairments and the severity of restricted and repetitive behaviors in ASD (54). Buch identified three latent dimensions of functional brain network connectivity that predicted individual differences in ASD behaviors (55). Our results provide further evidence that FC could predict social adaptability of ASD children at 4 years’ follow-up, involved brain regions including occipital cortex, paracentral lobule, temporal cortex, amygdala, superior frontal gyrus, parietal cortex (**Figures 4B, C**).

Structural covariance network (SCN) was introduced to explore the network level alterations (56), representing the covariance of morphological characteristics between regions, reflecting anatomical correlations in brain structure between brain regions (56–58). Previous researches about SCN based on gray matter density manifested that ASD showed greater covariance between right posterior cingulate cortex and right temporal compared with typically developmental controls (56, 59). In this study, we introduced individualized differential SCN (IDSCN), aiming to explore the heterogeneity of ASD from a structural perspective using SCN constructed at the individual level. Research mapped IDSCN based on Autism Brain Imaging Data Exchange (ABIDE) database to identify structural covariances, and found that IDSCN of ASD differed significantly from controls mainly involved frontal and subcortical regions (60). However, the research about relationship of the IDSCN and outcomes of ASD is few, our study fills this gap. In this study, we found IDSCN could also predict social adaptability of ASD children at follow-up, mainly involved thalamus, hippocampus, temporal cortex (**Figure 4B**). Further studies are needed to investigate the underlying mechanisms by which the brain predicts social adaptability of ASD.

### 4.3 Neuroimaging genetics findings on the outcomes of children with ASD

We explored the relationship of EFRS, brain connectivity and behaviors of ASD children, and found that ASD EFRS could not independently affect autism symptoms, but through the mediation of the FC of middle occipital gyrus (MOG) and pallidum (PAL) (see **Figure 5**), indicating that FC of MOG and PAL may be involved the neuroimaging mechanism of social communication symptoms of ASD caused by polygenic genetic risk. Previous brain imaging studies have also revealed evidence for genetic variations in brain activities underlying behavior, for example, it has been found that both local network metrics of the right hippocampus and its functional connectivity with the basal ganglia and thalamus mediated the relationship between the oxytocin receptor gene and interdependence (61); our results provide additional evidence of mediation role of the FC of MOG and pallidum in the genetics of ASD. Likewise, our findings further suggest a pathway from EFRS to behaviors of ASD, mediated by the FC from MOG to the pallidum. This result indicates that FC of the MOG and pallidum has a genetic basis and that the MOG and pallidum are crucial to the predictive prognosis of children with ASD.

Due to rapid developments in genomics and imaging technologies, neuroimaging genetics studies of ASD have developed in the last few years. Neuroimaging genetics helps to identify ASD-risk genes that contribute to structural and functional variations in brain of ASD patients (55, 62, 63), providing a better understanding of the disorder’s neuropsychiatry, and helping identify targets for therapeutic intervention that could be useful for the clinical management of ASD patients. Approaches of integrating neuroimaging and genetics to link gene pathways to neurobiological and phenotypic heterogeneity can reveal subtype-specific gene-brain-behavior associations (64). Recent research leveraged this method to identify three robust dimensional biomarkers, that was gene, brain and behaviors, to parse heterogeneity in ASD into 4 subgroups (55, 65). Within each subgroup, ASD-related FC was explained by regional differences in the expression of distinct ASD-related gene sets (55). These studies provide evidence that polygenic variation in ASD may manifest as intermediate behavior-related brain circuits that give rise to distinguishable ASD subgroup phenotypes by modulating connectivity in ASD-related networks. However, neuroimaging genetics studies about prognosis of ASD is limited, this motivates future research evaluating the reproducibility, validity, and clinical utility of ASD dimensional and subtype models in the outcomes of ASD. In the long term, there is translational potential for prognosis and targeted pharmacological and circuit-based therapies for ASD.

## 5 Limitations

Limitations of this study should be noted. Firstly, although this is a longitudinal follow-up study, we did not acquire the MRI data at follow-up, missing the predicting value of neuroimaging trajectory on the outcomes of ASD children. Secondly, our participants were limited to individuals with high-functioning ASD, as well as the limited cohort size, the explanation and generalization of the results in this study must be cautious. ASD is a heterogeneous neurodevelopmental disorder, showing great differences in terms of genetic predisposition, brain imaging, behaviors, and cognitive functions, so it is necessary for future studies to expand sample size to precisely identify neuroimaging genetics predictive biomarkers of prognosis of ASD children. In addition, this is a single cite study, so it is difficult to replicate across datasets. The last but not the least, we did not control the interventions of the affected children in this study, most of whom received non-systematically short-term, not qualified social and behavior interventions, thus, it is unlikely true that intervention factors have effects on the outcomes. In the future, larger cohort from multiple study cites, and more rigorous study design such as giving thought to interventions are needed to confirm our results.

## 6 Conclusions

Both EFRS and brain connectivity especially FC can predict the social adaptability outcomes of ASD children. The FC of left middle occipital gyrus and left pallidum mediates the relationship of EFRS and social communication of ASD, suggesting that occipital gyrus and pallidum play an important role in the etiology of ASD exome polygenic genetic risk and may be the intervention targets in the prognosis of children with ASD. Our findings could improve understanding of the neuroimaging genetics of ASD and suggest potential intervention targets to improve the outcomes of children with ASD.

## Data availability statement

The datasets presented in this study can be found in online repositories. The names of the repository/repositories and accession number(s) can be found in the article/**Supplementary Material**.

## Ethics statement

The studies involving humans were approved by Medical Ethical Committee of West China Hospital of Sichuan University. The studies were conducted in accordance with the local legislation and institutional requirements. Written informed consent for participation in this study was provided by the participants’ legal guardians/next of kin.

## Author contributions

TL: Conceptualization, Writing – original draft. MZ: Visualization, Writing – review & editing. SL: Data curation, Writing – review & editing. MS: Conceptualization, Writing – review & editing. PL: Investigation, Writing – review & editing. MW: Investigation, Writing – review & editing. YT: Conceptualization, Writing – review & editing. SZ: Investigation, Writing – review & editing. ZW: Investigation, Writing – review & editing. YY: Methodology, Writing – review & editing. YH: Conceptualization, Funding acquisition, Writing – original draft.
