# Supplementary material for: Exome functional risk score and brain connectivity can predict social adaptability outcome of children with autism spectrum disorder in 4 years’ follow up
Source: Front Psychiatry. 2024 May 16;15:1384134. doi: 10.3389/fpsyt.2024.1384134 (PMC11137745; doi:10.3389/fpsyt.2024.1384134)
Supplement: Supplementary file 1 [file DataSheet_1.docx]

Supplementary Materials

# 1 MRI data preprocessing and brain connectivity network construction

Raw data acquired from the MRI scanner (DICOM files) were converted from raw DICOM files into analyzable NIfTI images using dcm2niigui software. Use the SPM software package in MATLAB R2013b platform (https://www.fil.ion.ucl.ac.uk/spm/software/spm12/), FreeSurfer package (https://www.freesurfer.net/) and FSL software (http://surfer.nmr.mgh.harvard.edu/fswiki/Fsl) to analyze imaging data processing.

## T1 data processing

Preprocessing: In this study, SPM12 and FreeSurfer software packages were used to preprocess T1w images of all subjects, and the steps were as follows: 1. Quality control: An experienced imaging technician performed quality control to remove images with significant artifacts. 2. Normalize: Normalize to MNI standard space. 3. Modulate: The volume changes of the image are corrected by linear and nonlinear changes. 4. Smooth: the image was processed by Gaussian smoothing with 8mm×8mm×8mm full width at half maximum (FWHM). 5. Segment: T1w data were segmented to obtain gray matter, white matter and cerebrospinal fluid. Each image was carefully checked on the basis of automatic correction. Any segmentation errors were manually corrected by an experienced imaging technologist, and then the segmentation was re-calculated.

In this study, individual differential structural covariance network (IDSCN) was constructed to analyze the common morphology changes among the brain regions of the subjects. This method has been used in the field of brain imaging research in patients with schizophrenia(1). The main steps of constructing the IDSCN for each subject were as follows: 1). Construct the reference network: a reference structure covariance network was constructed for the entire control group (n controls) by calculating the correlation (partial Pearson correlation coefficient, PCC) of gray matter volume of each brain region, taking age, sex, education level, and total intracranial volume (TIV) as covariates. The reference structural covariance network was represented as PCC_n_. 2). Construct the perturbed network: we added patient k to the control group, and used n + 1 subjects (n controls and 1 patient) to construct a new structural covariance network, which we termed as the perturbed network PCC_n+1_. 3). The difference between the perturbed network and the reference network was calculated, i.e., ΔPCCn = PCC_n+1_ – PCC_n_. 4). The IDSCN network for patient k was then constructed with the weight of each edge was the Z-scores. The edges in the IDSCN represent how the additional patient changed the covariance of pairs of brain regions in their gray-matter volume against the reference group. We further obtained the P value for each edge in the IDSCN network for patient k from the Z-score. The atlas template used in this study is Automated Anatomical Labeling (AAL). a digital atlas that partitions the brain according to the information provided by the MNI institution, which was proposed by Tzourio-Mazoyer et al in 2002(2). The number of IDSCN nodes per subject was 90 (the number of brain regions in the AAL template). The edges obtained in the IDSCN represented how the covariance between the two connected nodes of a specific patient deviates from the normal covariance observed in the unaffected control group, that is, the deviation from the normal covariance of any two nodes.

## 1.2 Resting state functional MRI (rs-fMRI) data processing

The SPM12 software package was used for all rs-fMRI data preprocessing procedures. The specific steps include: 1) Slice timing: Discarded the first 10 time points to ensure the stability of the magnetic field; 2) Realign: Participants whose average head motion displacement was more than 2mm were excluded; 3) Normalize: All images were normalized to MNI space, and the voxel size was 3*3*3 mm^3^ after normalization. 4) Smooth (detrend): Gaussian smoothing (FWHM = 8mm) was applied to the normalized images; 5) Filter: Low frequency full band filter is used for filtering; 6) Regression: Regression of whole brain, white matter, cerebrospinal fluid signals, and Friston-24 head motion parameters.

The whole brain functional connectivity method based on correlation analysis proposed by Salvador (2005) was used for the construction of brain functional network (FC)(3): The average fMRI signal was extracted from the 90 brain regions of the AAL template as the nodes, and the Pearson correlation coefficient between the 90 brain regions was calculated after preprocessing. The correlation matrix was transformed into a normal distribution by Fisher-Z transformation to obtain the edges of the FC network.

# 2 Whole exome sequencing and calculating exome functional risk score

## 2.1 Steps of the whole exome sequencing

The main steps of sequencing are as follows: 1) Quality inspection: The quality of DNA samples was tested to detect whether there is obvious DNA degradation and whether there was RNA and protein contamination; 2) Library construction: DNA samples with content above 0.6ug of Agilent SureSelect Human All Exon V6 liquid capture system were effectively enriched to create sequence libraries; 3) Sequencing: high-throughput and deep sequencing was performed on the Illumina HiSeq 4000 platform (Illumina, Inc., San Diego, CA, USA) with 100 bp read length.

The preprocessing steps of whole exome sequencing data include extraction, quality control and typing annotation. 1) Extraction: Sequencing data were mapped onto human reference genome (GRCh37/hg19) using Burrows-Wheeler Aligner (BWA) software. Then, we used the filtering standard software program SAMtool and Picard (http://broadinstitute.github.io/picard/), which had been recognized internationally, to sort and perform repeated labeling on the bam files of 59 ASD subjects to generate the final bam file. Finally, perform HaplotypeCaller command of GATK (The Genome Analysis Toolkit) to extract mutations, a total of 9,286,6288 base pairs extracted. 2) Quality control: Quality control of the original file in base, gene and individual level (FastQC). Firstly, check the quality of base (Phred score > 30, leaving in only variants with base call accuracy of 99.9%), duplicate sequence (mapping quality Phred score > 20) and GC bias. Genes with genotyping quality score <20, read depth < 8X were excluded. We also excluded subjects having genotyping rate ≤ 95%, cryptic relatedness (identity by descent (IBD)>0.1875), abnormal heterozygosity and population outliers (outside five standard deviations from the mean for the first 20 population principal components). Individuals with known monogenetic conditions such as fragile X syndrome, tuberous sclerosis, and Rett syndrome were also excluded. In addition, we focused on the polygenic risk of rare mutations, so only mutations with minor allele frequency (MAF) <0.01, according to the east Asians MAF from the 1000 Genomes Project database (N=504) (4) and the Exome Aggregation Consortium (ExAC) database (N ~= 3000) (5), were retained for subsequent analysis. 3) Mutation typing and annotation: The mutations were annotated into 18 categories according to the recommendations of Human Genome Mutation Society. In this study, we only included nonsynonymous point mutations, that is frameshift, missense, stop gain, stop loss, start loss, and splicing.

## 2.2 Exome functional risk score

Polygenic risk score is a comprehensive assessment of the cumulative effects of multiple variants with weak effects on related diseases to evaluate the genetic risk of developing a disease (6). According to the procedure recommended by PRSice-2, the additive model was used to calculate the score (7). We followed the approach developed by Chiara Fabbri (8) to calculate ASD exome functional risk score (EFRS) based on the WES data, and to obtain the load scores of the whole exome rare variants. The EFRS was calculated using the following formula:

$$\sum_{1}^{n} v_{all}*w_{s}*w_{f}$$

where n is the number of variants within the entire exome, v_all_ is the number of alternative alleles, w_s_ is the corresponding functional score of the gene variant, and w_f_ is the frequency weight for that variant. By weighting function and frequency simultaneously, EFRS does not depend on the presence of individual variants which could not be observed in some of the tested samples, and thus keeps the final scores stable and reliable (9).

We adopted five pathogenicity prediction tools including LRT, Mutation Assessor, Polymorphism Phenotyping v2 (Polyphen-2), Sorting Intolerant From Tolerant (SIFT) and Combined Annotation Dependent Depletion (CADD) (10-13) to determine the damaging of mutations for functional scores (w_s_). 1) LRT: LRT was used for classification assignment: damaging, D=1; neutral, N =0; unknown, U= a (a is the probability of D among all alternative alleles). 2) Mutation Assessor (Mutation- Assessor.org///functional impact of protein mutations): The classification of functional impact of mutation in Mutation Assessor was used to assign values: functional high, medium, low and neutral were 1, 0.67, 0.33 and 0, respectively; with missing values U= a (missing values were filled with mean a). 3) Polyphen-2 (http://genetics.bwh.harvard.edu/pph2/): The Polyphen-2 prediction classification was assigned as follows: damaging, D = 1; possible damaging, P = 0.5; benign, B = 0; unknown, U=a (where a is the probability of D among all alternative alleles). 4) SIFT (https://sift.bii.a-star.edu.sg/): Individual amino acid changes are classified according to the function of the protein (Sequence Ontology): damaging, D=1; tolerant, T=0; unknown, U= a (where a is the probability of D among all alternative alleles). 5) CADD (https://cadd.gs.washington.edu/): All alternative alleles were assigned taxonomically using the adjusted CADD scores. In this study, we ranked and scored all 8.6 billion single nucleotide variations (SNVs) of the human reference genome (GRCh37/hg19), and then "PHRED scaled" these values by orders of magnitude. For example, the reference single nucleotide polymorphisms (SNPs) in the highest 10% of the CADD scores were assigned values of 10 or greater (“ ≥C10”), while variants in the highest 1%, 0.1%, etc. were assigned scores ≥ C20, ≥C30, etc. The adjusted CADD scores range from 1 to 25 after reassignment. The final functional scores achieved from the five prediction tools were compared and the best performing one was finally selected. The frequency weighting (w_f_) was determined based on the mean frequency of east Asian populations in the 1000 Genomes Project (https://www.internationalgenome.org/) and ExAc databases (http://exac.broadinstitute.org). The alternative alleles (v_all_) were determined based on the mutation sites of ASD identified in a WES study of 175 trios published in Nature in 2012 (14).

# Supplementary Figures and Tables

## 3.1 Supplementary Figures


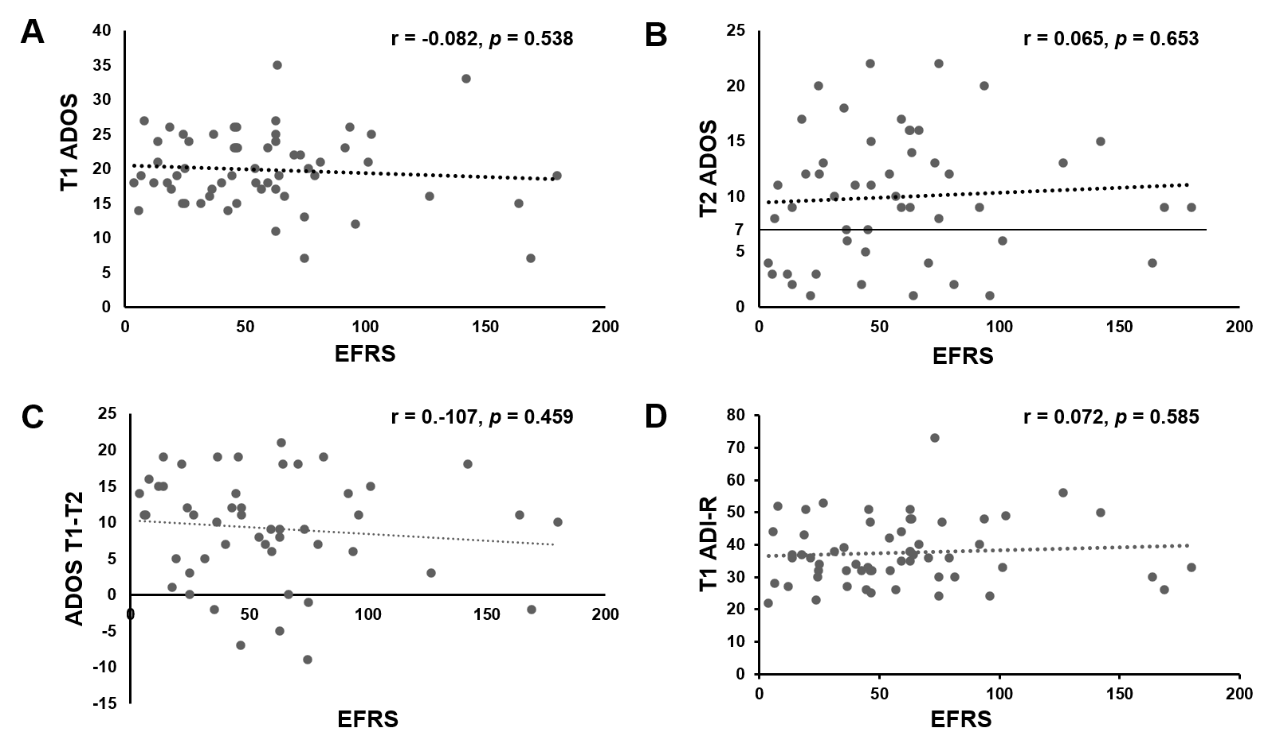


**Supplementary Figure 1.** Relationship of EFRS and phenotypes both in baseline and follow-up. **(A)** Relationship of EFRS and ADOS total scores at baseline (r = -0.082, p = 0.538); **(B)** Relationship of EFRS and ADOS total scores at follow-up (r = 0.065, p = 0.653); **(C)** Relationship of EFRS and reduction values of ADOS from baseline to follow-up (r = -0.107, p = 0.459); **(D)** Relationship of EFRS and ADI-R total scores at baseline (r = 0.072, p = 0.585).

Notes: The dashed line is the trend of the data. The solid line(B) represents the ADOS total score is 7; below the solid line are individuals with optimal outcome, not meeting the diagnostic criteria for ASD anymore; above the solid line are individuals with poor outcomes. EFRS, exome functional risk scores; ADOS, autism diagnostic observation schedule; ADI-R, the autism diagnostic interview-revised; T1, baseline; T2, follow-up.


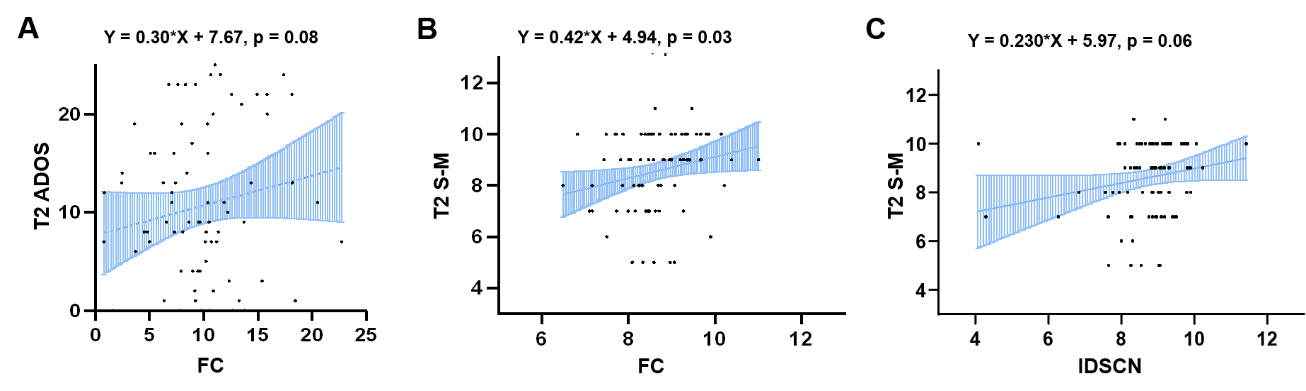


**Supplementary Figure 2.** The predictive effect of SVR models on outcomes of ASD children. **(A)** The predictive effect of SVR model on the ADOS total scores based on FC (fMRI), regression function: Y = 0.30*X + 7.67, R^2^ = 0.13, MSE = 79.25, p = 0.08; **(B)** The predictive effect of SVR model on the S-M based on FC (fMRI), regression function: Y = 0.42*X + 4.94, R^2^ = 0.13, MSE=2.33, p = 0.03; **(C)** The predictive effect of SVR model on the S-M based on IDSCN (sMRI), regression function: Y = 0.30*X + 5.97, R^2^ = 0.17，MSE = 2.66, p = 0.06. Notes: SVR, support vector regression; ADOS, autism diagnostic observation schedule; FC, functional connectivity; IDSCN, individual differential structural covariance network; fMRI, functional MRI; sMRI, structural MRI.


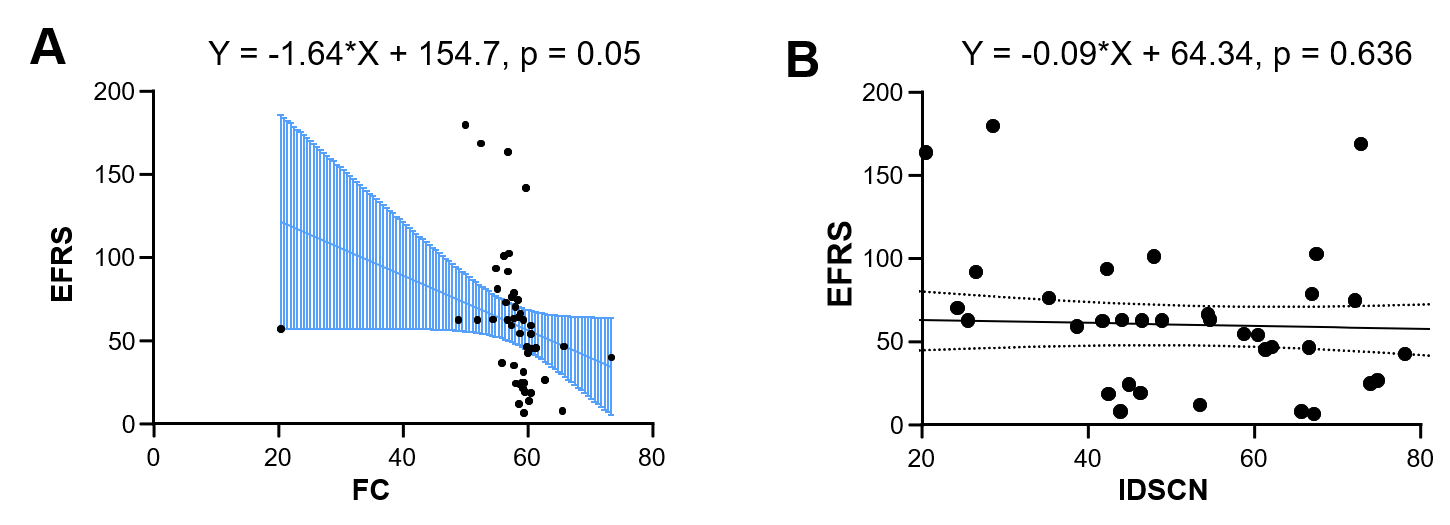


**Supplementary Figure 3.** The predictive effect of SVR models on ASD EFRS. (**A**) The predictive effect of SVR model on ASD EFPRS based on FC (fMRI), regression function: Y = -1.64*X + 154.7, *p* = 0.05, R = -0.32, MSE = 3168.80; **(B)** The predictive effect of SVR model on ASD EFRS based on IDSCN (sMRI), regression function: Y = -0.09X + 64.34, *p* = 0.636. Notes: FC, functional connectivity; IDSCN, individual differential structural covariance network; EFRS, exome functional risk score.

## 3.2 Supplementary Tables

**Supplementary Table 1**. Exome functional risk scores of 59 ASD children.

| score | ASD EFRS of 59 ASD children |
| --- | --- |
| EFRS | 19.21 62.79 73.09 24.79 59.26 101.19 62.72 21.43 42.72 141.99 46.38 74.75 126.61 78.98 40.10 66.39 3.82 36.24 13.76 6.52 179.84 163.78 70.42 45.36 7.87 44.40 23.80 63.39 46.62 11.89 168.81 13.64 62.66 95.94 81.24 17.58 26.59 56.91 24.92 74.69 54.11 64.04 93.54 59.31 31.39 5.57 35.32 46.74 36.71 91.67 18.58 46.14 24.33 62.84 54.48 76.23 62.67 45.52 102.53 |

Notes: EFRS, exome functional risk scores.

# Reference:

1. Liu Z, Palaniyappan L, Wu X, Zhang K, Du J, Zhao Q, et al. Resolving Heterogeneity in Schizophrenia through a Novel Systems Approach to Brain Structure: Individualized Structural Covariance Network Analysis. *Mol Psychiatry* (2021) 26(12):7719-31. doi: 10.1038/s41380-021-01229-4.

2. Tzourio-Mazoyer N, Landeau B, Papathanassiou D, Crivello F, Etard O, Delcroix N, et al. Automated Anatomical Labeling of Activations in Spm Using a Macroscopic Anatomical Parcellation of the Mni Mri Single-Subject Brain. *NeuroImage* (2002) 15(1):273-89. doi: 10.1006/nimg.2001.0978.

3. Salvador R, Suckling J, Coleman MR, Pickard JD, Menon D, Bullmore E. Neurophysiological Architecture of Functional Magnetic Resonance Images of Human Brain. *Cereb Cortex* (2005) 15(9):1332-42. Epub 2005/01/07. doi: 10.1093/cercor/bhi016.

4. Siva N. 1000 Genomes Project. *Nature biotechnology* (2008) 26(3):256. Epub 2008/03/11. doi: 10.1038/nbt0308-256b.

5. Lek M, Karczewski KJ, Minikel EV, Samocha KE, Banks E, Fennell T, et al. Analysis of Protein-Coding Genetic Variation in 60,706 Humans. *Nature* (2016) 536(7616):285-91. Epub 2016/08/19. doi: 10.1038/nature19057.

6. Bogdan R, Baranger DAA, Agrawal A. Polygenic Risk Scores in Clinical Psychology: Bridging Genomic Risk to Individual Differences. *Annual review of clinical psychology* (2018) 14:119-57. Epub 2018/03/27. doi: 10.1146/annurev-clinpsy-050817-084847.

7. Choi SW, O'Reilly PF. Prsice-2: Polygenic Risk Score Software for Biobank-Scale Data. *GigaScience* (2019) 8(7). Epub 2019/07/16. doi: 10.1093/gigascience/giz082.

8. Fabbri C, Kasper S, Kautzky A, Zohar J, Souery D, Montgomery S, et al. A Polygenic Predictor of Treatment-Resistant Depression Using Whole Exome Sequencing and Genome-Wide Genotyping. *Transl Psychiatry* (2020) 10(1):50. Epub 2020/02/19. doi: 10.1038/s41398-020-0738-5.

9. Lali R, Chong M, Omidi A, Mohammadi-Shemirani P, Le A, Cui E, et al. Calibrated Rare Variant Genetic Risk Scores for Complex Disease Prediction Using Large Exome Sequence Repositories. *Nat Commun* (2021) 12(1):5852. Epub 2021/10/08. doi: 10.1038/s41467-021-26114-0.

10. Adzhubei IA, Schmidt S, Peshkin L, Ramensky VE, Gerasimova A, Bork P, et al. A Method and Server for Predicting Damaging Missense Mutations. *Nature methods* (2010) 7(4):248-9. Epub 2010/04/01. doi: 10.1038/nmeth0410-248.

11. Ng PC, Henikoff S. Sift: Predicting Amino Acid Changes That Affect Protein Function. *Nucleic acids research* (2003) 31(13):3812-4. Epub 2003/06/26. doi: 10.1093/nar/gkg509.

12. Kircher M, Witten DM, Jain P, O'Roak BJ, Cooper GM, Shendure J. A General Framework for Estimating the Relative Pathogenicity of Human Genetic Variants. *Nat Genet* (2014) 46(3):310-5. Epub 2014/02/04. doi: 10.1038/ng.2892.

13. Ionita-Laza I, McCallum K, Xu B, Buxbaum JD. A Spectral Approach Integrating Functional Genomic Annotations for Coding and Noncoding Variants. *Nat Genet* (2016) 48(2):214-20. Epub 2016/01/05. doi: 10.1038/ng.3477.

14. Neale BM, Kou Y, Liu L, Ma'ayan A, Samocha KE, Sabo A, et al. Patterns and Rates of Exonic De Novo Mutations in Autism Spectrum Disorders. *Nature* (2012) 485(7397):242-5. Epub 2012/04/13. doi: 10.1038/nature11011.
